# Muscle synergies in preparation to a step made with obstacle in elderly individuals

**DOI:** 10.1186/s12984-015-0005-9

**Published:** 2015-02-04

**Authors:** Yun Wang, Kazuhiko Watanabe, Tadayoshi Asaka

**Affiliations:** Tianjin Key Lab of Exercise Physiology and Sports Medicine, Department of Health and Exercise Science, Tianjin University of Sport, 51 Weijin South Street, Hexi District, Tianjin, 300381 China; Institute of Sports and Health Science, 3-10-31, Kagamiyama, Higashi-hiroshima, Hiroshima, 739-0046 Japan; Department of Rehabilitation Science, Faculty of Health Sciences, Hokkaido University, N12-W5, Kita-ku, Sapporo, 060-0826 Japan

**Keywords:** Anticipatory synergy adjustments, Synergies, Step initiation, Obstacle, Elderly

## Abstract

**Background:**

To evaluate if multi-muscle synergies are comprised of flexible combinations of a small number of postural muscles to stabilize the center of pressure (COP) shift during preparation to making a step in the elderly (self-paced level stepping vs. obstacle crossing stepping).

**Methods:**

Electromyography (EMG) signals of leg and trunk muscles were recorded. Linear combination of integrated indices of muscle activity (M-modes) and their relationship to changes in the COP shift in the anterior-posterior (AP) direction were first determined. Uncontrolled manifold (UCM) analysis was performed to determine the extent to which variance of the M-modes acted to produce a consistent change in the COP displacement.

**Results:**

The elderly were capable of stabilizing the COP_AP_ coordinate based on co-varied involvement of the M-modes. The synergy index (∆V) changes in the elderly emerged later (100 ms prior to t_0_) and its magnitude was smaller as compared to that reported in younger persons.

**Conclusions:**

Our study reveals that aging is associated with a preserved ability to explore the flexibility of the M-mode compositions but a decrease ability to use multi-M-mode synergies following a predictable perturbation.

## Background

Stepping over an obstacle is a complex task that requires translating the center of mass closer to the edge of the base of support, with large inertial forces that could potentially threaten stability. Because the sensory and motor resources that are required for postural stability decline with age, stepping over an obstacle can become quite a demanding task and pose a great risk in older adults [1,2]. Moreover, aging is associated with major changes in the neuromotor system. In particular, these include reduction in muscle strength, power and joint mobility along with an impaired sensorimotor integration. All these changes can potentially contribute to deterioration of postural control and mobility in the elderly. Previous studies have shown that lower accuracy and higher variability in obstacle crossing tasks by elderly subjects [3,4].

Wang et al. investigated the multi-muscle synergies used by healthy young participants to stabilize the anterior-posterior (AP) trajectory of the center of pressure (COP) during preparation to making a step with obstacle [5]. Within the obstacle-negotiation paradigm, stepping over obstacle task from quiet stance was combined to comfortable level stepping. These different tasks were designed to study the different organizations of leg and trunk muscles into groups (M-modes) and trial-to-trial co-variations of M-mode involvement (M-mode synergies) during stepping tasks, using the uncontrolled manifold (UCM) analysis. The UCM analysis assumes that the neural controller acts in a space of independent elemental variables (for example, electromyographic signals, EMGs) and creates in that space a sub-space (UCM) corresponding to a value or a time profile of a specific performance variable (for example, COP trajectory), which is assumed to be important for postural tasks. It begins with identification of M-modes with parallel scaling of muscle activation levels [6-10]. Then, the COP shifts are mapped on small changes in the M-modes magnitudes, resulting in a Jacobian matrix. Further, co-variation of M-modes magnitudes is analyzed to quantify synergies stabilizing the COP coordinate [9]. Wang et al. found that most of the across-trials variance in the M-modes space stabilized the average value of COP_AP_ shift (“good variance”; within UCM, V_UCM_) whereas the component of the M-modes space variance (“bad variance”; orthogonal to UCM, V_ORT_) resulting in COP_AP_ variability was smaller. As such, these findings highlight the importance of M-modes in control of posture, and point out the existence of the robustness of multi-M-mode synergies across different manners of making a step.

While such coordination pattern in the activation of the lower extremities and the trunk muscles are established in the healthy young adults [5-10], to the best of our knowledge there are no studies that evaluate the effect of aging on the multi-M-mode synergies during the stepping over obstacle task. Given that aging is associated with a decline in muscle mass, strength, coordination and postural stability [11,12], comprehending the age-related differences in the muscle synergies during preparation to making a step with obstacle is vital. While the impairments of anticipatory postural control in the elderly have been demonstrated [13,14], it is not specifically known how these changes influence multi muscle coordination in controlling the body’s COP displacement closer to the boundaries of the base of support, that compromising balance. The outcome of a recent study on postural control in step initiation revealed that there were M-mode synergies stabilizing COP shifts in the stepping and supporting legs in the young and elderly subjects. However, the synergies of the older adults showed a reduced and delayed value than that of the young persons [15]. These findings indicate that when initiating gait from a quiet standing position, older adults may have difficult in utilizing anticipatory postural synergies. As such, it is important to investigate whether older adults are able to effectively utilize preparatory muscle coordination in balance maintenance prior to making a step with obstacle.

Therefore, the objective of the present study was focused on examining possible changes of multi-M-mode synergies during preparation to making a step in elderly individuals. We hypothesized that multi-M-mode synergies would be observed during the task of stepping over obstacle. In particular, we expected to see that the synergies of the older adults will be delayed and reduced in magnitude and will be associated with greater COP displacements in the anterior-posterior (AP) direction.

## Methods

### Subjects

Nine healthy older adults (five males and four females; mean age = 72.3 ± 4.4 yr, mean weight = 59.0 ± 9.2 kg, and mean height = 160.0 ± 5.2 cm) participated in the study. None of the subjects had a balance disorder or experienced dizziness, and none of them had neurological or musculoskeletal disorders. All of the subjects were right-foot-dominant according to preferred foot usage when kicking a ball, stepping up on a chair, and leaping off in the long jump [16]. All of the subjects gave their informed consent to take part in the study, which is consistent with the 1964 Declaration of Helsinki.

### Experimental protocol

**Figure 1:**
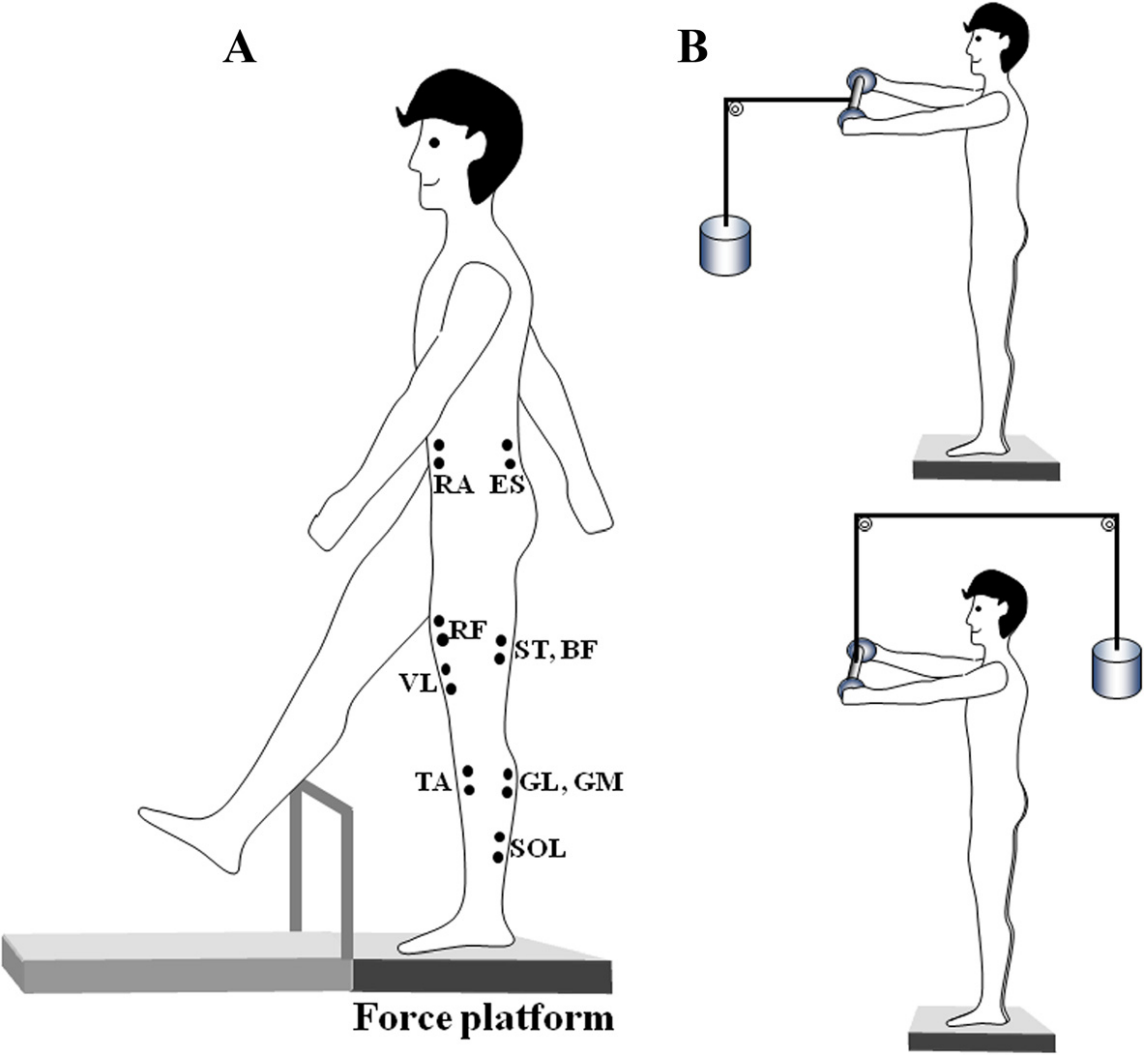
**The experimental setup.** **A**. The subjects were required to step over an obstacle of 15% body height from quiet stance in a self-paced way. **B**. In the control trials, the subjects were required to hold a load (5.3 Kg) in front of the body or behind the body for 5 s, they held a handle which was connected to the load through a pulley system. Location of some of the EMG electrodes is also shown in panel A (*GL* lateral head of gastrocnemius, *GM* medial head of gastrocnemius, *SOL* soleus, *ST* semi-tendinosus, *BF* biceps femoris, *GMED* gluteus medius, *ES* erector spinae, *TA* tibialis anterior, *VL* vastus lateralis, *RF* rectus femoris, *TF* tensor fasciae latae, *RA* rectus abdominis).

To test the effect of aging on the ability of older adults to change the multi-muscle synergies in preparation to a step made with obstacle, we presented subjects with an obstacle set at 15% of the subject’s body height (Figure 1, A). A lightweight PVC pipe crossbar was used as the obstacle. The choice of the obstacle height was based on the following considerations. This task was used in our earlier studies of obstacle crossing [5,17,18] and the same high obstacle height on older subjects has been used in previous researches [2,3] to reflect typical high height encountered in everyday life. The obstacle location was chosen in any distance by each subject.

The experiment included two parts (*control-test* and *stepping-test*). The *control-test* involved two tasks (Figure 1, B and C): (1) quiet standing while holding steadily a load of 5.3 kg in front of the body through the pulley system (*QS*_*LF*_*task*); (2) quiet standing while holding steadily a load of 5.3 kg behind the body (*QS*_*LB*_*task*) through the pulley system. When the subjects were facing the pulley, they counteracted the load by activating the dorsal muscles of the leg and trunk muscles; when they were facing away from the pulley, the ventral muscles were activated. For each task, the subjects were required to stand as still as possible and keep the body vertical for 5 s. The *control-test* was used to normalize the EMG signals for individual subjects. The *stepping-test* involved two tasks: (1) comfortable stepping task (*ST*_*CS*_), (2) obstacle stepping task (*ST*_*OS*_). Before the *stepping-test,* 2-5 practice trials were given to all subjects for familiarization with the task.

The *stepping-test* required the subjects to step forward with the right leg and followed with the left leg so that both feet came to rest forward. In the *ST*_*CS*_ task, the subjects were asked to make a comfortable level step from quiet stance in a self-paced manner. In the *ST*_*OS*_ task, the subjects were required to step over an obstacle of 15% body height from quiet stance in a self-paced way [5]. For each task, the subjects were instructed to look straight ahead and with no restrictions to the arms. The tasks were performed in two blocks of 20 trials. There were at least a 6-s interval between trials and a 2-min interval between tasks, to avoid fatigue. Foot position was marked on the top of the platform to keep the same position across all the trials. The tasks were performed in a random order across subjects.

### Data collection

In all tasks, the subjects stood barefoot on a force platform (AMTI, Watertown, MA; Model BP400600-2000) with their feet shoulder-width apart, eyes open. The vertical component of the ground reaction force (F_Z_), the horizontal component of the ground reaction force in the anterior-posterior direction (F_X_), and the moment of force about the frontal-horizontal axis (M_Y_) were recorded. Electromyography (EMG) activity was recorded from ten lower limb and trunk muscles of the subject’s right side. After the skin was shaved and cleaned with alcohol, active surface EMG electrodes (Biometrics, United Kingdom) were placed on the muscle bellies. The following muscles were recorded: tibialis anterior (TA), lateral head of gastrocnemius (GL), medial head of gastrocnemius (GM), soleus (SOL), rectus femoris (RF), vastus lateralis (VL), biceps femoris (BF), semitendinosus (ST), rectus abdominis (RA) and erector spinae (ES). Experimental data were digitized at the sampling frequency of 1000 Hz with a 16-bit resolution. A foot switch was placed under the heads of the metatarsal bones of right foot to measure the timing of toe off.

### Data processing

The data were processed offline using MATLAB Version 8.0 (R2012b, The MathWorks, Natick, MA) software packages. Raw EMG data were rectified and filtered using fourth-order Butterworth low-pass filter with a 50-Hz cutoff frequency. Signals from the force plate were low-pass filtered at 20 Hz. Coordinates of the COP in the anterior-posterior (AP) direction were calculated based on the formula:

1 $$ \mathrm{C}\mathrm{O}{\mathrm{P}}_{\mathrm{AP}}=\left(\hbox{-} {\mathrm{M}}_{\mathrm{Y}}+\left({\mathrm{F}}_{\mathrm{X}}\times \mathrm{d}\right)\right)/{\mathrm{F}}_{\mathrm{Z}} $$

where coefficient *d* is the distance from the origin of the force platform to its top surface (0.045 m according to the manufacturer’s specifications) [19].

The ‘time zero’ (t_0_) was defined by the toe off time using the signal from the foot switch. Rectified EMG signals were integrated over 25 ms intervals in a time window from -500 ms (before t_0_) to t_0_. These EMG integrals for each of 25 ms were then corrected by subtracting the EMG integrals of the baseline activity during quiet neutral stance in the control trial. The outcome of the adjusted EMG integral will be denoted as *I*EMG. ∆*I*EMG indices were further normalized (∆*I*EMG_N_) by the EMG integrals collected in the control trials as follows: ∆*I*EMG indices for the dorsal (SOL, GL, GM, BF, ST, and ES) muscles were divided by the EMG integrals over 25 ms (*I*EMG_C_) in the middle of the control trial with holding the load quietly in front of the body, while ∆*I*EMG indices for the ventral (TA, RF, VL, and RA) muscles were divided by the EMG integrals over 25 ms (*I*EMG_C_) in the middle of the corresponding control trial, that is during holding the load quietly behind the body. Five 100 ms time windows in relation to t_0_ were analyzed, from -500 to -400 ms (T1), from -400 to -300 ms (T2), from -300 to -200 ms (T3), from -200 to -100 ms (T4) and from -100 ms to t_0_ (T5). Different time intervals were defined based on our prior studies to reflect possible time development of the multi-muscle synergies [5,7].

### Defining M-modes and Jacobians

We extracted groups of muscles (M-modes) from the *I*EMG_N_ data matrix within the time window in relation to t_0_ from -200 ms to t_0_ using PCA. For each subject, the *I*EMG_N_ data formed a matrix of 8 time intervals × 10 muscles × 20 trials =1600 data points. The correlation matrix among the *I*EMG was subjected to principal component analysis with Varimax rotation, using procedures from SPSS (SPSS, Inc., Chicago, Illinois, USA). The factor analysis module with principal component extraction was employed.

For each subject, the obtained eigen-values and PCs were then considered. The first four PCs (described in more detail in Results) were selected for further analysis. This was determined by examining of the scree plots and having at least two muscles significantly loaded per PC. We are going to address these PCs as muscle modes (M-modes) and assume that magnitudes of (coefficients at) the M-modes are manipulated by the controller to produce COP_AP_ shifts. A reciprocal M-mode is defined as a pattern with significant loading coefficients on the ventral muscles (“push-back” mode), or on the dorsal muscles (“push-forward” mode), while a co-contraction M-mode is defined as a pattern with significant loading coefficients on the same M-mode with the same sign for two muscles with opposing action at a particular joint [8].

Small changes in the magnitudes of M-modes (∆M) were related to the change in the COP_AP_ shifts (∆COP_AP_) through the Jacobian (**J**). Multiple linear regression analysis over the trials was used to define the **J** for each subject separately. The **J** was estimated as coefficients of multiple linear regression between across-trials ∆Ms and ∆COP_AP_.

### Computing synergy index: UCM analysis

For each trial of the *ST*_*CS*_ and *ST*_*OS*_ tasks, ∆IEMG_N_ were computed and transformed into ∆Ms by multiplying the loadings of the individual M-mode. The mean magnitudes of each ∆M for a selected time interval across a series of ST trials were computed. Since the model relating ∆Ms to ∆ COP_AP_ is linear, the mean values were subtracted from each computed value and the residuals were further analyzed.

The UCM represents different combinations of M-modes that keep the value of ∆ COP_AP_ unchanged. The UCM was estimated as the null space of the corresponding **J** matrix. The null space is spanned by the basis vectors, **ɛ**_*i*_. The vector of individual mean-free ∆Ms was resolved into its projection onto the null space and the orthogonal subspace:

2A $$ {f}_{UCM}={\displaystyle {\sum}_{i=1}^{n-d}\left({\varepsilon}_i^T\cdot \left(\varDelta M\right)\right)}\;{\varepsilon}_i $$

2B $$ {f}_{ORT}=\left(\varDelta M\right)-{f}_{UCM} $$

where n = 4 and d = 1are the number of dimensions of the UCM and of task space.

Variance per degree of freedom within the UCM and orthogonal to the UCM across trials were computed as:

3A $$ {V}_{UCM}={\sigma}_{UCM}^2={\displaystyle {\sum}_{i=1}^N{f}_{UCM\;}^2}/\left(\left(n-d\right){N}_{trials}\right) $$

3B $$ {V}_{ORT}={\sigma}_{ORT}^2={\displaystyle {\sum}_{i=1}^N{f}_{ORT}^2}/\left(d{N}_{trials}\right) $$

We computed an index of synergy (Δ*V*) reflecting the difference between the variance within the UCM and orthogonal to the UCM:

4 $$ \varDelta V=\left({V}_{UCM}-{V}_{ORT}\right)/{V}_{TOT} $$

where all variance indices are computed per degree of freedom; *V*_TOT_ means the total variance. For further analyses, the Δ*V* values were transformed using a Fisher’s z-transformation (Δ*V*_*Z*_) adapted to the boundaries of Δ*V*:

5 $$ \Delta {V}_Z=\frac{1}{2}\cdot log\left[\frac{4+\Delta V}{1\frac{1}{3}-\Delta V}\right] $$

### Statistics

The data are presented in the text and figures as mean ± SD. The fractions of variance explained by the first four principal components were transformed into z-scores using standard Fisher’s z-transformation. Paired *t* test was used for comparing the z-scores and the peak COP_AP_ shifts between the two tasks. For the data of ΔV_Z_, two-way ANOVA was used with factors *Task* (ST_CS_ and ST_OS_), *Time interval* (five intervals) to analyze possible changes in values of ΔV_Z_ across tasks and with time. Tukey post-hoc tests were used where appropriate. For all statistical analyses, p-value less than 0.05 was set as a measure of significance.

## Results

### General EMG patterns and COP displacements

**Figure 2:**
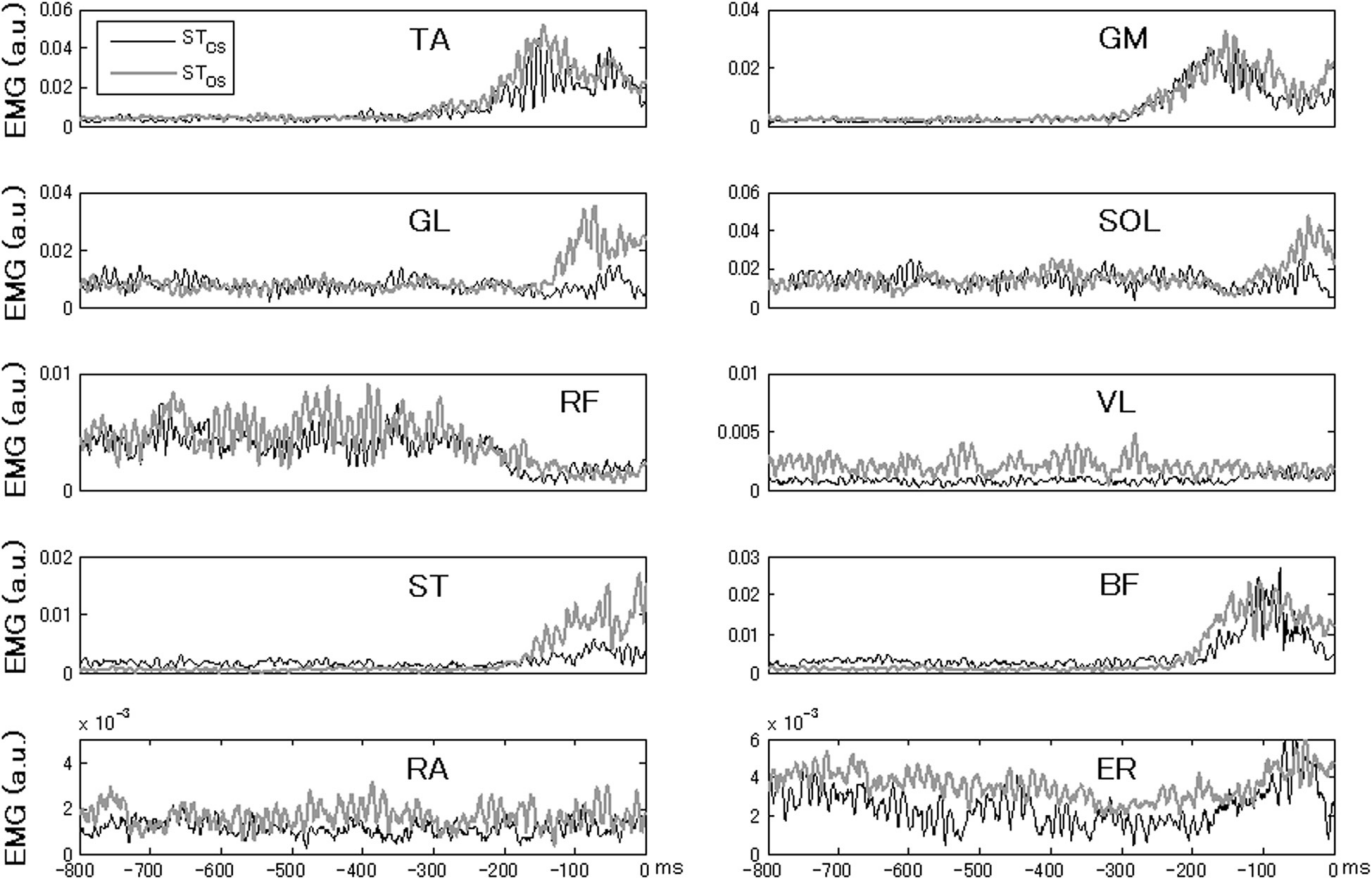
**Typical EMG patterns averaged across twenty trials by a representative subject for the ST**_**CS**_ **(dark lines) and ST**_**OS**_ **(gray lines) tasks.** Time zero corresponds to the alignment time, the time of toe off. Note the early increase in the activity of dorsal muscles, accompanied sometimes by a decrease in the activity of ventral muscles. The EMGs were recorded in muscles of the right side of the body. The EMG scales are in arbitrary units and time is in ms.

Postural muscles demonstrated similar early changes across the EMG patterns associated with making a step. Figure 2 shows the rectified EMGs of the leg and trunk muscles averaged across trials by a representative subject in the ST_CS_ and ST_OS_ tasks. In the ST_CS_ task, the stepping leg typically showed alternating bursts of activity in the ventral and dorsal muscles. The ventral muscles showed a decrease in the baseline activity but the dorsal muscles showed an increase in the activity just before the step initiation. In the ST_OS_ task, there was a substantial increase in the level of activity of most muscles. The regularities in the patterns of activation of the leg and trunk muscles were consistently observed in the ST_CS_ and ST_OS_ tasks. Note that muscle activity in the ST_CS_ task was typically higher than that in the ST_OS_ task. Muscle activity varied across subjects, and some subjects did not show clear bursts or episodes of EMG suppression in some muscles.

**Figure 3:**
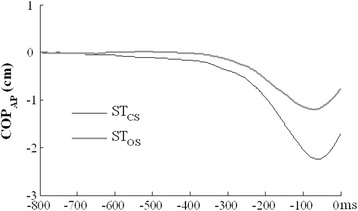
**Average COP**_**AP**_ **displacement is shown for a representative in the ST**_**CS**_ **and ST**_**OS**_ **tasks.** Negative values correspond to backward displacements. Dark and gray lines indicate the ST_CS_ and ST_OS_ tasks, respectively.

In preparation to stepping, subjects shifted the COP in the AP direction backwards (Figure 3). This adjustment allowed to unload the stepping leg and to create a moment of the reactive force rotating the body forward about the ankle joints. There was larger anticipatory COP_AP_ displacement in the ST_CS_ task (COP_AP-CS_ = -2.42 ± 1.22 cm) as compared to the ST_OS_ task (COP_AP-OS_ = -1.75 ± 1.02 cm); the difference was statistically significant (p < 0.05). Negative values correspond to backward displacements.

### Principal component analysis and multiple regression analysis

Indices of integrated muscle activity were measured within the time window in relation to t_0_ from -200 ms to t_0_ (see the Methods). The normalized integrated EMG indices (*I*EMG_N_) were subjected to principle component analysis (PCA) with Varimax rotation to identify muscle groups, eigenvectors in the muscle activation space. As a result, the ten-dimensional muscle activation space was reduced to a four-dimensional M-mode space.

**Figure 4:**
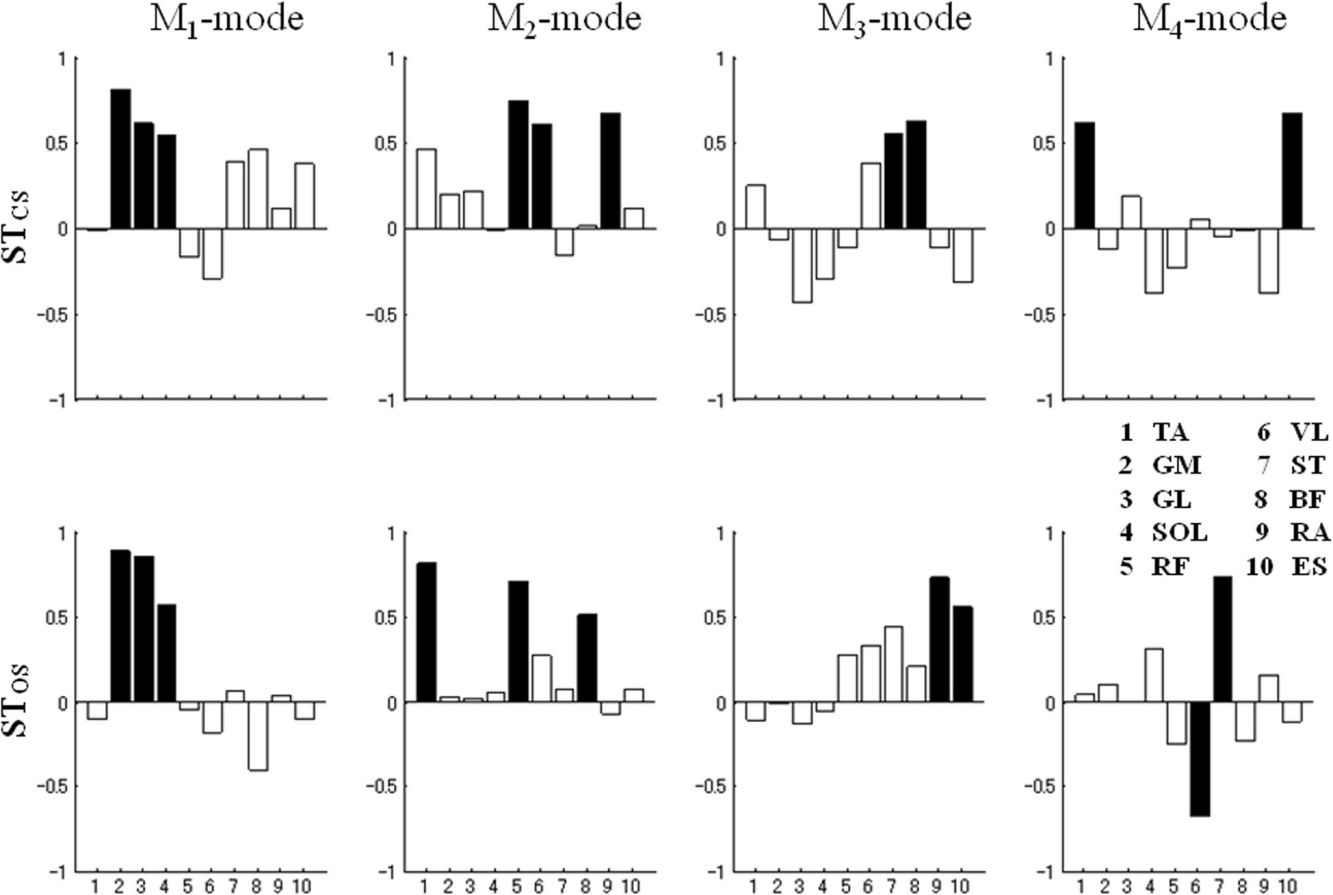
**Loading coefficients for the PCA of the ST**_**CS**_ **and ST**_**OS**_ **tasks for a representative subject.** Loading magnitudes over | ± 0.5| are shown in black (significant loadings).

On an average, the first four principal components (PCs) accounted for the 62.2 ± 5.9% total variance in the muscle activation space in the ST_CS_ task and 60.0 ± 4.2% in the ST_OS_ task. There was considerable variability across the subjects in the M-mode composition. The loadings for all the muscles on the four factors for a representative subject in the ST_CS_ task are presented in Figure 4 (top panel). The first M-mode showed high loading values with the same sign for the IEMG_N_ indices of the GL, GM, SOL muscles. The second M-mode showed high loading values for the TA, RF, VL muscles. The muscles which showed high loading in the third M-mode were the ST and BF muscles. In the fourth M-mode however, the loading pattern were higher for the RA and ES muscles.

It is important to note that the first M-mode revealed a reciprocal contraction of the leg muscles (“push-back” M-mode) and the second M-mode depicted a reciprocal contraction of the thigh and leg muscles (“push-forward” M-mode). Similarly, significant loading coefficients for the third M-mode revealed a reciprocal contraction of the thigh muscles (“push-back” M-mode). A reciprocal M-mode is defined as a pattern with significant loading coefficients on the ventral muscles (“push-back” mode), or on the dorsal muscles (“push-forward” mode). Significant loading coefficients for the fourth M-mode seen in the RA and ES muscles with opposing actions revealed a co-contraction at the hip joint.

Representative results of the PCA in the ST_OS_ task are presented in Figure 4 (bottom panel). The first M-mode composition was a “push-back” M-mode. The second M-mode showed high loading values for two ventral (TA and RF) and one dorsal (BF) muscles acting at the knee joint, while the third M-mode showed high loading values for the RA (ventral) and ES (dorsal) muscles, which is a “co-contraction at the hip” pattern. The fourth M-mode again showed a push-back pattern between the VL and ST muscles, with the opposite sign. Overall, in the ST_CS_ task, 10 M-modes with “co-contraction” M-mode were seen, while in the ST_OS_ task, the number of M-modes with co-contraction patterns was 15 from a total of 36 M-modes.

Multiple regression analysis was performed to define the Jacobian mapping small changes in the M-mode magnitudes onto COP_AP_ shifts. Results of multiple regression analysis were significant in most cases for each of the two tasks. On an average, the analysis accounted for 85.7 ± 10.2% and 85.0 ± 14.7% of variance in ∆COP_AP_ in the ST_CS_ and ST_OS_ tasks respectively. There was no task difference in variations in the magnitudes of the four M-modes accounted for the total variance in ∆COP_AP_ (*P* > 0.05).

### Synergy analysis

The muscle coordination pattern that elderly subjects used to stabilize the COP_AP_ shift was examined by measuring the index of multi-M-mode synergy (∆V) using the UCM analysis. The synergy index was computed as the normalized difference between the variance within the UCM and orthogonal to the UCM. Positive ∆V values indicate that most variance within a given time window was within the UCM, i.e. that an average value of COP_AP_ displacement observed within that window was stabilized by co-variation of magnitudes of the M-modes.

**Figure 5:**
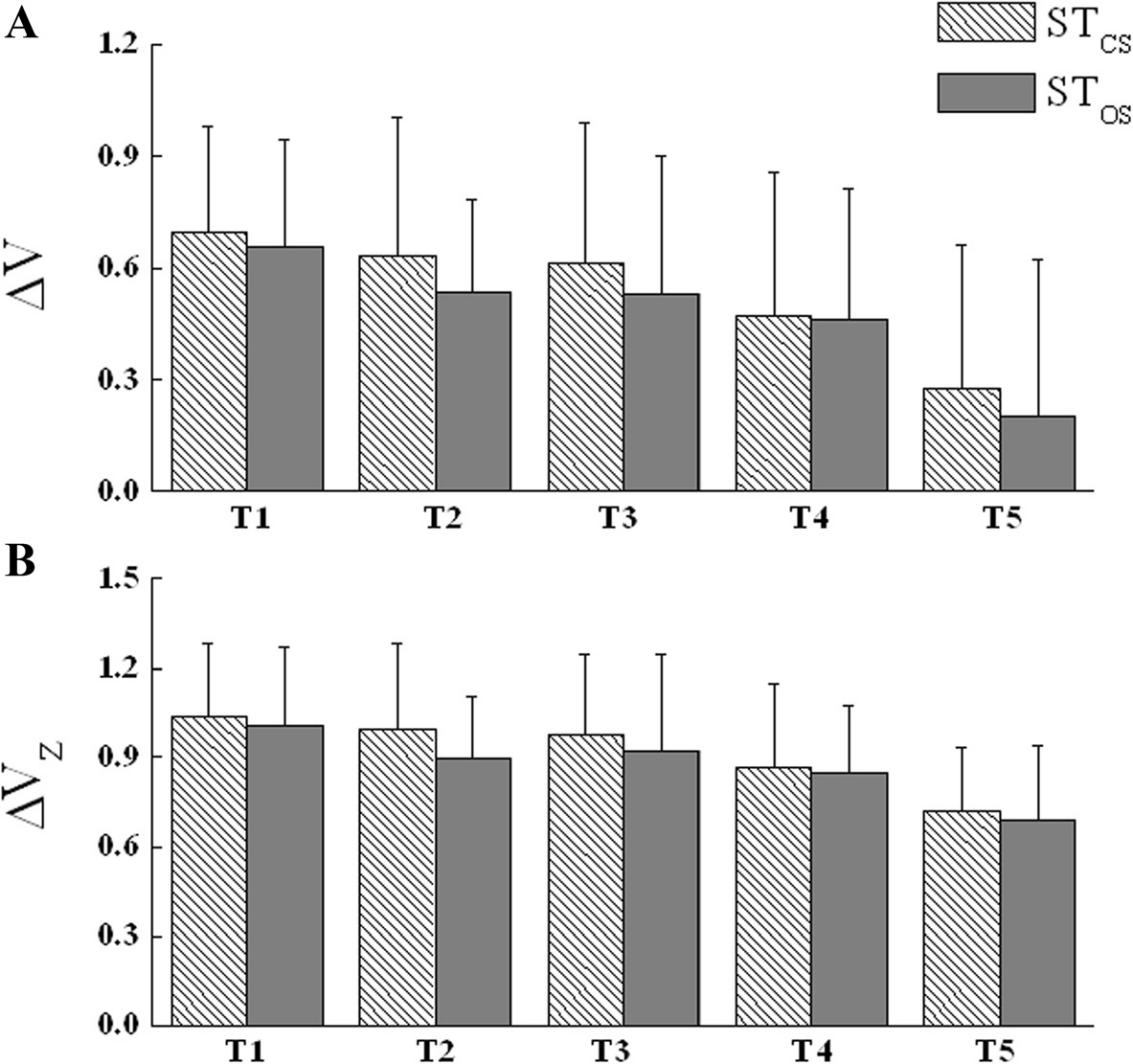
**Mean across subjects ± standard deviation of ∆V (panel A) and ΔV**_**Z**_ **(panel B) indices for the control of the COP**_**AP**_ **shift.** Adjacent pairs of bars represent the ST_CS_ (left, stripped bars) and ST_OS_ (right, gray filled bars) tasks. ∆V indices and ΔV_Z_ indices were averaged over five 100 ms time intervals starting 500 ms prior to t0 and ending up at t0.

Figure 5A shows ∆V indices averaged across subjects computed for COP_AP_ shifts during the ST_CS_ and ST_OS_ tasks. In general, subjects demonstrated multi-M-mode synergies stabilizing COP_AP_ shifts (∆V > 0). They all showed reproducible time changes for both ST tasks, there were relatively minor differences in ∆V between the ST tasks. A two-way ANOVA with the factors *Task* (ST_CS_ and ST_OS_) and *Interval* (T1, T2, T3, T4 and T5) was performed to analyze possible differences in the ΔV_Z_ value (Figure 5B). The ANOVA showed significant main effect of Interval [F_(4,64)_ = 10.194, *P* < 0.01], whereas there were no significant main effect of Task [F_(1,16)_ = 0.228, *P* > 0.05]; no interaction was observed between Interval and Task [F_(4,64)_ = 0.183, *P* > 0.05]. About 100 ms prior to the time of toe off (time zero), there was a drop in ΔV_Z_ seen across tasks. Post-hoc analyses revealed that ΔV_Z_ were significant greater for T1, T2, T3, T4 than ΔV_Z_ for T5 (*P* < 0.05).

## Discussion

The main finding of the experiment is that, when stepping over an obstacle, the older adults were capable of stabilizing the COP_AP_ coordinate based on co-varied involvement of the M-modes. The synergy index (∆V) changes in the elderly emerged later (100 ms prior to t_0_) and its magnitude was smaller as compared to that reported in younger persons. This result indicates that aging is associated with a preserved ability to explore the flexibility of the M-mode compositions but a decrease ability to use multi-M-mode synergies following a predictable perturbation.

Several recent studies suggested that postural stability involved multi-muscle synergies, especially when performing whole-body actions [7-9,20,21]. Those studies confirmed that the existence of multi-muscle synergies in a variety of everyday actions associated with the activities of daily living (ADL). The purpose of synergies has been assumed to ensure adequate mechanical conditions for maintaining optimal postural control in upright stance. Stepping over an obstacle is not an ADL but it may hamper ADL. A decline in obstacle crossing performance with advancing age may be implicated in the higher incidence of trips and stumbles in older adults [22]. Due to the changes in postural control with age [13,23-26], it is feasible that age-related changes in multi-muscle synergies can lead to trip-related falls. Yet much less attention has been paid to the ability to utilize synergic multi-muscle control of vertical posture in older persons.

When making a step with obstacle, coordinated changes in anticipatory activation of the leg and the trunk muscles are observed prior to the stepping foot takeoff. In the current study, a reciprocal M-mode was seen in the distal leg muscles of older adults in both the ST_CS_ and ST_OS_ tasks. Within this M-mode, dorsal muscles were generally activated whereas ventral muscles were inhibited. In the ST_OS_ task, the proximal muscles showed more of co-contraction M-mode at the knee and hip joints. This finding suggests that the older adults chose to use a reciprocal strategy to control the ankle joint while increase the stiffness of the other joints for stabilizing the COP displacement when dealing with the instability. Indeed, similar strategies of increasing joint stiffness with co-contraction of thigh and trunk muscles under challenging postural conditions have been previously reported in healthy young and old individuals [5,8,12,13,15,20,21,25,27,28] and in individuals with neurological disorders [29,30] who deliberately use muscle co-contraction trading efficacy for safety. Taken together with the literature, the observed adjustments in the composition of M-modes reflect the preserved ability of elderly persons to explore the flexibility of the mechanically redundant multi-muscle system and find different solutions for compensating for the declined ability to maintain upright posture.

The coordination of multiple muscles about the longitudinal axis of the body contributes to postural stability has been shown to involve the use of muscle redundancy by the central nervous system (CNS) [5,7-10]. These studies suggest that the CNS takes advantage of muscle redundancy to stabilize the COP success by allowing for flexible combinations of redundant degrees of freedom. It should be noted that the capability to produce varied solutions to a postural task leads to movement variability. Successful postural performance requires its variability has no effect on the performance variable under consideration. The use of the uncontrolled manifold (UCM) analysis allows to separate M-mode variance into a component that reflects flexibility in stabilizing the COP (“good” variance within the UCM subspace) from a component that leads to variability of the COP (“bad” variance within the orthogonal subspace). As such, UCM analysis quantifies the structure of movement variability. The index of M-mode co-variation (∆V) reflects the relative amounts of “good” and “bad” variance in the M-modes space. If the synergy index (∆V) is close to ‘+1.33’ (see equation 4 in the Methods), most M-mode variance reflects the use of muscle redundancy to stabilize the COP_AP_ shift [31]. In the current study, the pattern of ∆V change was similar to a pattern reported in a similar study of young subjects [5]. However, in the study of younger persons, the magnitude of ∆V drop was significant 200 ms prior to the time of heel off of the stepping leg and its magnitude was about +0.6. In the current study, ∆V changes in the elderly emerged later (100 ms prior to t_0_) and their magnitude (+0.2) was about one-third of that reported in younger persons. The findings of smaller and delayed anticipatory synergy adjustments (ASAs) resemble closely the observations of preparation to a step made under the reaction time instruction in the elderly [15]. In both studies, ASAs to a self-triggered perturbation could be generated by the elderly subjects, but these adjustments were smaller and closer in time to heel off. These results suggest that although ASAs are delayed and reduced in magnitude with aging, the ability to utilize multi-M-mode synergies is largely preserved during preparation to making a step with obstacle.

Our experimental design had an inherent limitation of the number of EMG channels we could record simultaneously. We have decided to record muscles only on the stepping leg. It is possible that the supporting leg could also be important for giving valuable information which may not have been accounted for considering the changes in stepping strategy in elderly when making a step with obstacle. We will try to overcome it in future studies.

## Conclusions

Postural adjustments prior to step initiation with obstacle represent a particular example of anticipatory actions; understanding adjustments on such actions to changes in the external conditions would be important for better understanding the anticipatory motor control processes with aging. Our study reveals that aging is associated with a preserved ability to explore the flexibility of the M-mode compositions but a decrease ability to use multi-M-mode synergies following a predictable perturbation. The results provide a foundation for investigating the role of training in improving the composition of M-modes and patterns of their co-variation with respect to important performance variables of postural control in older adults.

## Abbreviations

AP: Anterior-posterior
BF: Biceps femoris
COP: Center of pressure
ES: Erector spinae
GL: Lateral head of gastrocnemius
GM: Medial head of gastrocnemius
PCA: Principal component analysis
RA: Rectus abdominis
RF: Rectus femoris
SOL: Soleus
ST: Semitendinosus
TA: Tibialis anterior
UCM: Uncontrolled manifold
VL: Vastus lateralis
